# Experimental Protection of Diabetic Mice against Lethal *P. aeruginosa* Infection by Bacteriophage

**DOI:** 10.1155/2014/793242

**Published:** 2014-06-05

**Authors:** Nagaveni Shivshetty, Rajeshwari Hosamani, Liyakat Ahmed, Ajay Kumar Oli, Syed Sannauallah, Shivshetty Sharanbassappa, S. A. Patil, Chandrakanth R. Kelmani

**Affiliations:** ^1^Department of Biotechnology, Gulbarga University, Gulbarga, Karnataka 585106, India; ^2^Luqman College of Pharmacy, Gulbarga, Karnataka 585101, India; ^3^Department of Pathology, M. R. Medical College, Gulbarga, Karnataka 585104, India; ^4^Department of Neuromicrobiology, NIMHANS, Bangalore, Karnataka 560 029, India

## Abstract

The emergence of antibiotic-resistant bacterial strains has become a global crisis and is vulnerable for the exploration of alternative antibacterial therapies. The present study emphasizes the use of bacteriophage for the treatment of multidrug resistant * P. aeruginosa*. * P. aeruginosa* was used to induce septicemia in streptozotocin (STZ) induced diabetic and nondiabetic mice by intraperitoneal (i.p.) injection of 3 × 10^8^ CFU, resulting in a fatal bacteremia within 48 hrs. A single i.p. injection of 3 × 10^9^ PFU phage GNCP showed efficient protection in both diabetic (90%) and nondiabetic (100%) bacteremic mice. It was further noted that the protection rate was reduced in diabetic mice when phage GNCP was administered after 4 h and 6 h of lethal bacterial challenge. In contrast, nondiabetic bacteremic mice were rescued even when treatment was delayed up to 20 h after lethal bacterial challenge. Evaluation of results confirmed that a single intraperitoneal injection of the phage dose (3 × 10^9^ PFU/mL) was more effective than the multiple doses of imipenem. These results uphold the efficacy of phage therapy against pernicious * P. aeruginosa* infections, especially in cases of immunocompromised host.

## 1. Introduction

After decades of extensive use of antibiotics in the treatment of infectious diseases caused by pathogenic bacteria, the emergence of multidrug resistant bacterial strains combined with a slowdown in the discovery of new classes of antibiotics is currently viewed as a major public health concern.* Pseudomonas aeruginosa* is the predominant pathogen that causes severe nosocomial diseases such as septicemia, pneumonia, and urinary tract infection in immunocompromised individuals [1].* P. aeruginosa* has now become a major cause of nosocomial infections due to its remarkable propensity to rapidly acquire resistance determinants to a wide range of antibacterial agents [2]. Diabetes mellitus affects several aspects of the immune system. Functional properties of polymorphonuclear leukocytes, monocytes, and lymphocytes such as adherence, chemotaxis, and phagocytosis are depressed in patients with diabetes [3]. Other alterations in the immune system may include reduced cell-mediated immune responses, impaired pulmonary macrophage function, and abnormal delayed type hypersensitivity responses. The risk of recurrence of such infections is also higher in diabetic patients. Some specific types of infections also occur predominantly in diabetic patients (malignant otitis externa, rhinocerebral mucormycosis, emphysematous pyelonephritis and cholecystitis, and Fournier's gangrene) [4]. The prevalence of* P. aeruginosa *in the impaired immune system candidates leads to the complications of secondary infections. Infections of* P. aeruginosa *in diabetic patients are bacteremia, sepsis, emphysematous pyelonephritis, skin and soft tissue infections, and more frequent in malignant otitis external disease. Of note* P. aeruginosa *has a greater ability to develop resistance to virtually any antibiotic to which it is exposed, because of multiple resistance mechanisms that can be present within the pathogen. Therefore, the development of the alternative antibacterial approach is necessary for the treatment of a broad array of antibiotic-resistant infectious diseases. Bacteriophages or phages are viruses that specifically infect and lyse bacteria. A method of using phage for the treatment of bacterial infectious disease is called bacteriophage therapy or phage therapy. Recently, phage therapy has gained an increasing attention because it has many advantages over chemotherapy. Phages have high specificity for their target bacteria, indicating that they do not harm the normal intestinal microflora. Phages are effective against multidrug resistant pathogenic bacteria because the mechanisms by which they induce bacteriolysis differ completely from those of antibiotics. Moreover, phage has self-limitation, meaning that the number of phages remains at very low level after killing the target bacteria [5].

The following study, was employed to examine the efficacy of phage therapy in abrogating fatal* P. aeruginosa* infection in diabetic and nondiabetic mice models.

## 2. Materials and Methods

### 2.1. Bacterial Strains

*P. aeruginosa *strains were isolated from diabetic patients using BHI agar, and positive isolates were selected for future studies. Standard strain* P. aeruginosa* ATCC 27853 was used as a control.

### 2.2. Bacteriophage Isolation and Purification

*P. aeruginosa *strains were used as hosts to isolate specific phage from raw sewage. Phage isolations were accomplished by adding salt (58 g of NaCl) to 1 liter of sewage, followed by centrifugation at 12,000 rpm for 10 min. The supernatant was decanted into a separate sterile container and mixed with polyethylene glycol (PEG 8,00) to provide a final PEG concentration of 10% (w/v). The PEG containing supernatant was precipitated overnight at 4°C and centrifuged at 14,000 rpm for 20 min. The resulting precipitate was dissolved in 5 mL of phage dilution buffer (SM) and extracted once with an equal volume of chloroform. An aliquot (200 *μ*L) of this processed sewage was mixed with 100 *μ*L of an overnight culture of* P. aeruginosa *strain, incubated at 37°C for 20 min, mixed with 2 mL of molten top agar (0.8% agarose) at 50°C, and poured onto bacteriophage isolation agar plates (1.6% agar). Plates were incubated overnight at 37°C. Phage plaques were harvested from the plate, and single plaques were purified thrice on host strains.

### 2.3. Large Scale Amplification and Purification of Phage Particles

Phage was purified according to the procedure described by Biswas et al. (2002).* P. aeruginosa *host strains were suspended at 2 × 10^8^ cells/mL in 20 mL LB medium and were exposed to the crude preparation of GNCP phage and vigorously shaken for 4-5 h at 37°C, resulting in the complete lysis of bacteria. Later on treatment with 1% chloroform at 37°C for 10 min, followed by treatment with 1 *μ*g/mL DNase I and 1 *μ*g/mL RNase A for 30 min at 37°C, the culture fluid was centrifuged at 10,000 rpm for 10 min at 4°C to remove cell debris. PEG/NaCl was added to the supernatant to a final concentration of 1/6 (v/v) and was kept overnight at 4°C. The resultant precipitate containing the phage particles was collected by centrifugation at 10,000 rpm for 20 min at 4°C and resuspended in 500 *μ*L of SM buffer. The phage was collected and dialysed against 10 mM saline that contained 50 mM Tris-Chloride (pH 8.0) and 10 mM MgCl_2_ for 2 h at 4°C until used. The samples were approximately diluted with LB just before use. The titers (for infections) (PFU/mL) of purified samples were determined by inoculating them into* P. aeruginosa *strain [6].

### 2.4. Characterization of Phage

#### 2.4.1. Transmission Electron Microscopy

To observe the morphology, transmission electron microscopy (TEM) was performed by the modified method [6]. A drop of purified phage suspension was applied to former carbon-coated copper grid for five min. The suspension was removed with a pipette and negatively stained with 2% uranyl acetate (TAAB Laboratory, UK). After ten minutes the grids were examined in a Tecnai Biotwin (Philips), transmission electron microscope (The Netherlands).

#### 2.4.2. Bacteriophage DNA and Restriction Enzyme Analysis

Purified phage particles (2 × 10^8^ PFU/mL) were treated with 1 *μ*g of DNase I and RNase A (Bangalore Genei, Bangalore, India) at 37°C for 1/2 h. To the mixture, proteinase K (Bangalore Genei, Bangalore, India) and SDS were added at a final concentration of 0.05 mg/mL and 0.5%, respectively, and incubated at 56°C. After 1 h of incubation, an equal volume of phenol: chloroform was added to remove proteinaceous material. The extraction was repeated thrice with phenol: chloroform: isoamyl alcohol (25 : 24 : 1). The nucleic acid was precipitated with chilled ethanol and suspended in 20 *μ*L of TE buffer (10 mM Tris-HCl, pH 7.0, 1.0 mM EDTA, pH 7.0) according to standard procedure.

Restriction enzyme digestion of the isolated phage DNA was carried out following the instructions supplied by manufacturers. Hind III, Eco RI, and Bam HI were added to the purified bacteriophage DNA.

### 2.5. Experimental Animals

Specific pathogen free, colony bred, virgin adult Swiss mice (Wistar strain) of both sexes with the commendation of the Institute Animals Ethics Committee (Reg. number 346/CPSCEA) of Luqman Pharmacy college, Gulbarga, Karnataka, India, were obtained. Animals were fed standard pellet and water ad libitum. Each group of mice received an intraperitoneal injection of 60, 120, 150, and 180 mg of STZ (kg^−1^ of body weight). Control mice received citrate buffer (pH 4.5) alone. Blood glucose levels were monitored over the stabilization period of 10 days following administration of STZ by blood glucose monitors drawing blood from the tail vein [7].

### 2.6. Experimental Induction of* P. aeruginosa *Bacteremia in Diabetic and Nondiabetic Mice

For each infection experiment, 6- to 8-week-old diabetic and nondiabetic mice were divided into two groups, and each group was given inocula of various sizes. The infecting bacteria were prepared by growing* P. aeruginosa *in LB broth medium, at 37°C, and were centrifuged at 8000 rpm for 5 min. The cell pellet was washed with normal saline (0.9 gm in 100 mL distilled water), centrifuged again under the same conditions, and finally resuspended in 10 mL saline. After appropriate dilution, turbidity at 600 nm was measured to determine bacterial cell numbers. To find the minimal lethal dose (MLD), serial dilutions of* P. aeruginosa *(10^7^, 10^8^, and 10^9^ CFU/mL) were injected intraperitoneally (i.p.) into both diabetic and nondiabetic mice in 100 *μ*L aliquots. After infection, mice were kept under standard laboratory conditions with free access to food and water. Six mice were used for each dose; the survival rates of nondiabetic and diabetic mice were then measured at 2 days after infection [8]. Mice inoculated with* P. aeruginosa* were observed for several clinical signs, including ruffled fur, hunchback moribund, and partially closed eyes.

### 2.7. Imipenem Treatment in* P. aeruginosa *Infected Mice

Followed by intraperitoneal injection of* P. aeruginosa*, both diabetic and nondiabetic mice were randomly separated into four groups. The first two diabetic and nondiabetic groups received no antibiotic; the remaining groups received intraperitoneal injections of imipenem [30 mg (kg^−1^ of body weight)] after 20 min of pathogen administration. An antibiotic was given once daily for 5 days. The mice were observed for 30 days after the completion of treatment to determine the effect of antibiotics on infected diabetic and nondiabetic mice [9].

### 2.8. Treatment of Diabetic and Nondiabetic Bacteremic Mice with Phage

Ability of phage GNCP to rescue mice was seen in two groups using diabetic and nondiabetic* P. aeruginosa *DPA-12 bacteremic mice model. The effect of phage dose was studied in the six groups of mice (six mice in each group) and was challenged by intraperitoneal injection of* P. aeruginosa *DPA-12. Each of these groups was treated with a single injection of GNCP administered i.p. 20 min after the bacterial challenge at 3 × 10^10^, 3 × 10^9^, 3 × 10^8^, 3 × 10^7^, 3 × 10^6^, and 0 PFU. The state of the death of the mice was monitored for 30 days.

The result on the outcome of delaying treatment for various periods was also monitored. In the delayed-treatment study, treatment was initiated at 0, 1, 2, 3, 4, and 6 h after bacterial challenge with the MLD. The state or health of diabetic and nondiabetic bacteremic mice was monitored for 30 days.

### 2.9. Chronic Studies on STZ Induced Diabetic Mice

Chronic study was carried out in normal mice and streptozotocin induced diabetic mice for 21 days. The blood glucose levels of the animals were checked after 18 hrs of fasting and were considered as a 0 day reading. The phage 3 × 10^9^ dose was given daily to the animal for 21 days. Glipizide was used as a standard control. The blood levels were checked at 0-, 7-, 15-, and 21-day period. Blood glucose levels were monitored by drawing blood from the tail vein [7].

### 2.10. Determination of Immunologic Response to Phage GNCP in Mice

Indirect enzyme-linked immunosorbent assay was used to check phage GNCP-specific immunogenicity for IgG and IgM antibody titers in sera of diabetic and nondiabetic mice as described [6].

### 2.11. Histopathological Studies

With the intention to know the histological changes during the course of therapy by using bacteriophage as immunogens in mice, the careful gross examination of spleen, liver, kidney, and lung was done and dissected out. Samples preserved in 10% formalin were dehydrated in an ascending series of alcohol (70–100%). The tissue was embedded in paraffin wax, sectioned, and stained with hematoxylin and eosin [10].

## 3. Results

### 3.1. Isolation and Characterization of Bacteriophage

The phage isolated in the present study was found to form plaques on* P. aeruginosa *clinical isolate. A total of 12* P. aeruginosa *isolates from diabetic samples were used for isolation of lytic bacteriophage. The isolated phage was found to form plaques on four imipenem susceptible clinical isolates and also inhibited bacterial growth of two imipenem resistant strains including DPA 12. The phage GNCP showed it was able to multiply very rapidly on a* P. aeruginosa *DPA-12 culture, approximately reaching counts of 10^8^ PFU/mL within 2 h at 37°C. Electron microscopy revealed that phage GNCP has an icosahedral-shaped head, approximately 50 nm, and a nonrigid tail. Based on the morphology, the phage is tentatively placed in the Siphoviridae family (Ackermann, 2001). The phage DNA was 21 Kb in size. Of the four restriction enzymes (Hind III, EcoRI, BamHI, and Sma I) which were tried on bacteriophage, EcoRI was found to produce the clearest pattern of bands (data not shown).

### 3.2. Diabetes Induction in Experimental Mice

Each group of mice received an intraperitoneal injection of 60, 120, 150, and 180 mg of STZ (kg^−1^ of body weight), while control mice received citrate buffer alone. Mice given multiple doses of STZ [150 mg (kg^−1^ body weight)] showed marked and persistent hyperglycemia. Some mice received 180 mg of STZ (kg^−1^ body weight) and died within 15 days, but almost all mice given 150 mg of STZ (kg^−1^ body weight) were alive for more than 2 months. Although diabetic mice exhibited glycosuria, they developed a ruffled thinner appearance and consumed more water per day than the untreated group. Mice with fasting blood glucose levels >250 mg/mL were defined as diabetic mice and used as STZ induced mice model in the following experiments.

### 3.3. Selection of* P. aeruginosa *Strain to Induce Experimental Bacteremia in Diabetic and Nondiabetic Mice

Among 12 clinical isolates of* P. aeruginosa, *DPA-12 was selected on the basis of multidrug resistance including imipenem and its susceptibility for phage GNCP lytic multiplication. Hence,* P. aeruginosa *DPA-12 was selected for induction of bacteremia in diabetic and nondiabetic mice.

### 3.4. Induction of Experimental Bacteremia in Diabetic and Nondiabetic Mouse Model with* P. aeruginosa *DPA-12

**Figure 1:**
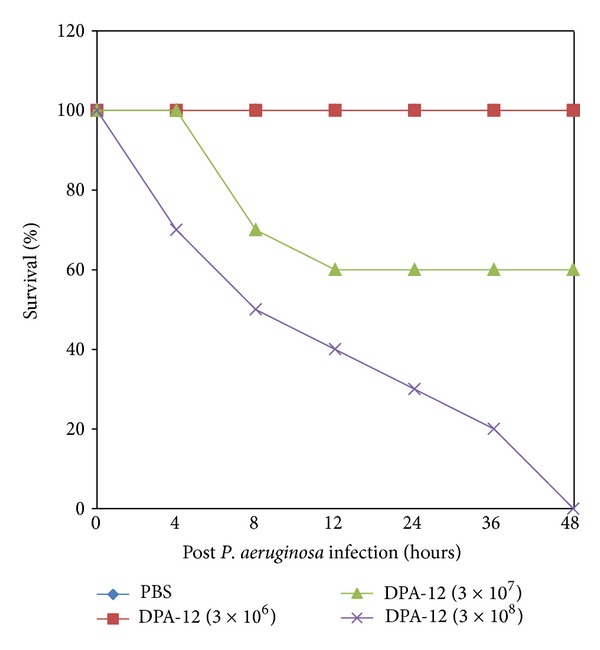
Determination of the MLD of* P. aeruginosa *(DPA-12) in nondiabetic mice. 25 in each group were infected i.p. with serially diluted suspensions of* P. aeruginosa*. The percentage of survival was determined up to 48 h.

**Figure 2:**
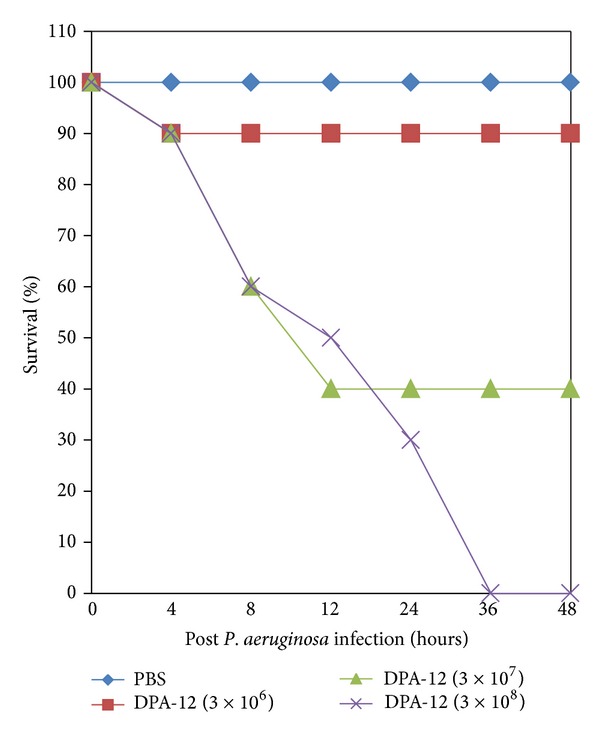
Determination of the MLD of* P. aeruginosa *(DPA-12) in diabetic mice. 25 in each group were infected i.p. with serially diluted suspensions of* P. aeruginosa*. The percentage of survival was determined up to 48 h.

The lethal dose of* P. aeruginosa *in mice was determined by injecting both diabetic and nondiabetic mice with varying numbers of* P. aeruginosa *DPA-12, ranging from 3 × 10^4^ to 3 × 10^9^ cells per dose. Intraperitoneal injections of 3 × 10^4^ to 3 × 10^6^ DPA-12 did not reduce the survival rate of nondiabetic mice whereas 10% diabetic mice died during the subsequent 7-day observation period. In contrast, injections of* P. aeruginosa* DPA-12 of 3 × 10^7^ cells showed survival rate of 60% in nondiabetic and 40% in diabetic mice within 48 h, whereas 3 × 10^8^ CFU/mL of* P. aeruginosa* DPA-12 showed 100% lethal effect within 48 h of injection in both groups of animals (Figures 1 and 2); therefore this dose 3 × 10^8^ CFU/mL was considered to be optimal and fixed throughout the experiment.

### 3.5. Efficacy of Phage against Lethal Bacteremia in Diabetic and Nondiabetic Mice

**Figure 3:**
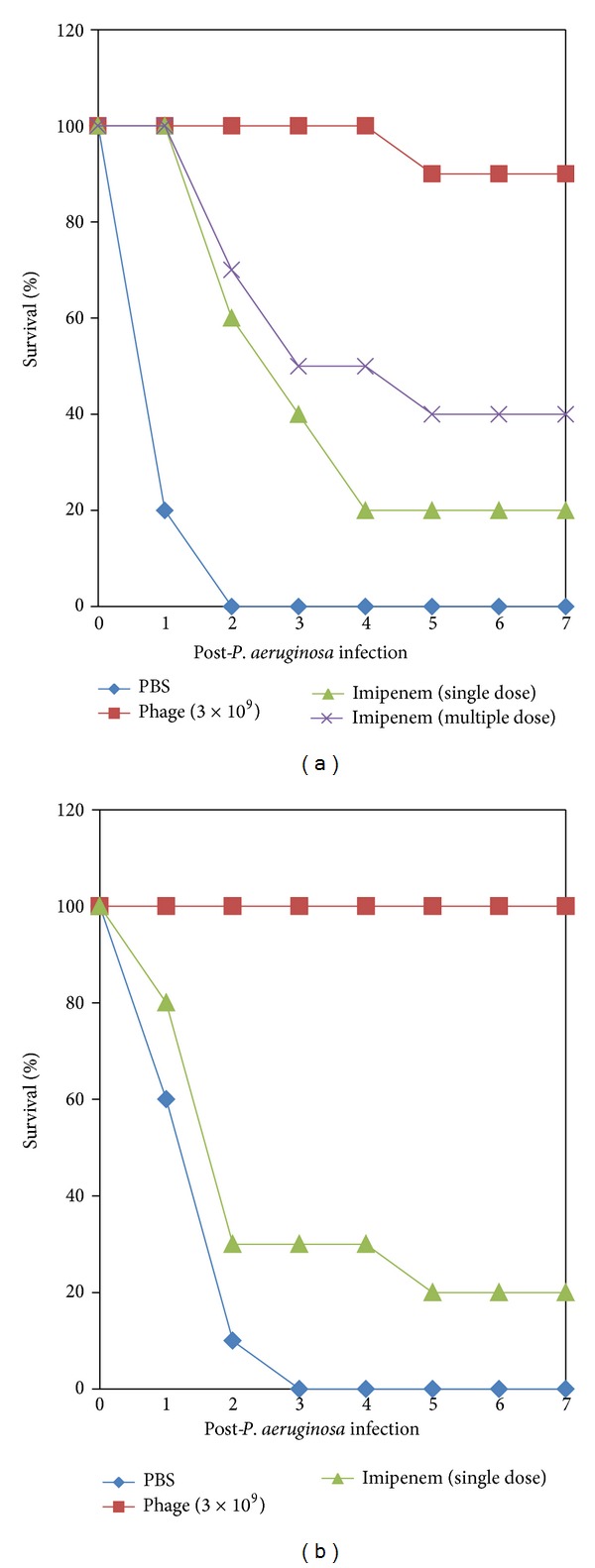
(a) The protection efficacy of phage therapy with imipenem (single and multiple dose) treatment of diabetic bacteremic mice. The percentage of survival was determined up to 7 days following i.p. challenge. (b) The protection efficacy of phage therapy with imipenem (single dose) treatment of nondiabetic bacteremic mice. The percentage of survival was determined up to 7 days following i.p. challenge.

Administration of a single dose of purified phage GNCP of 3 × 10^6^ to 3 × 10^8^ PFU protected up to 90% of diabetic mice and 90% of nondiabetic mice from DPA-12-induced lethal bacteremia. Phage dose of 3 × 10^9^ PFU/mL significantly rescued 90% of diabetic and 100% of nondiabetic mice from lethal bacteremia. All live mice remained healthy for an additional 30 days of observation. At this point the experiment was terminated. The phage dose effect on the state of health of the infected animals was clearly visible. Administration of a high dose (3 × 10^9^) of phage GNCP alone to experimental mice did not affect their physical condition or survival during the one-month period of observation (Figures 3(a) and 3(b)).

### 3.6. Phage Treatment Compared with Imipenem Treatment in Diabetic and Nondiabetic Bacteremic Mice

On comparing the protective efficacy of phage therapy with chemotherapeutic treatment of diabetic and nondiabetic bacteremic mice, a single i.p. dose of imipenem [(30 mg (kg^−1^ body weight)] showed 20% protection of diabetic bacteremic mice, whereas three consecutive injections of imipenem protected 40% of diabetic mice from* P. aeruginosa *DPA-12 bacteremia (Figure 3(a)). However, a single dose of imipenem treatment rescued 20% of nondiabetic bacteremic mice as shown in Figure 3(b). The administration of a single dose of phage GNCP of 3 × 10^9^ PFU rescued 90% and 100% of diabetic and nondiabetic mice from* P. aeruginosa *bacteriemia, respectively. The experimentally protected mice were healthy and active for the subsequent 7-day observation period. There were no survivors among untreated mice (in both groups) 2 days after intraperitoneous injection of 3 × 10^8^ CFU cells of* P. aeruginosa *DPA-12 (*P* > 0.02).

### 3.7. Effect of Delay in Treatment on the Ability of the Phage GNCP to Protect Diabetic and Nondiabetic Mice from* P. aeruginosa *DPA-12 Bacteremia

**Figure 4:**
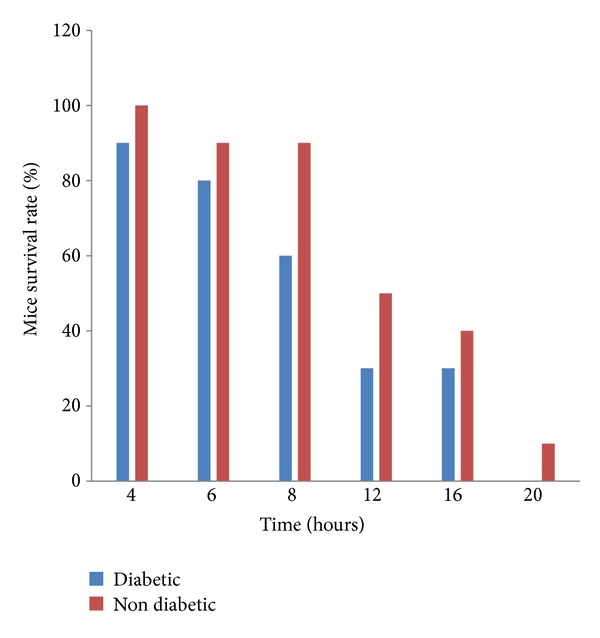
Delayed phage treatment of diabetic and nondiabetic bacteremic mice. A single i.p. injection of 3 × 10^9^ PFU was administered to bacteremic mice at the indicated times after bacterial challenge. Delayed phage administration rescued significantly higher numbers of nondiabetic bacteremic mice than diabetic bacteremic mice.

After induction of bacteremia in diabetic and nondiabetic mice with a lethal dose of* P. aeruginosa *DPA-12, phage therapy was applied through a single injection of 3 × 10^9^ PFU of phage GNCP at various intervals thereafter, ranging from 4 to 20 h. The experiments revealed that a single injection of phage (3 × 10^9^ PFU) can rescue 90% and 100% of diabetic and nondiabetic bacteremic mice, respectively, even when treatment is delayed for 4 h after i.p. injection of lethal dose of* P. aeruginosa *DPA-12 of bacterial challenge (Figure 4). A delay in phage administration by 6 and 8 h led to decreased protection rates, 80% and 60%, respectively (*P* > 0.03), in diabetic bacteremic mice. In comparison, nondiabetic bacteremic mice were rescued 90%, 90%, and 40%, when the treatment was delayed up to 6, 8,  and 16 h, respectively, after lethal dose of bacterial infections (*P* > 0.05). When treatment was delayed beyond 20 h, only 10% of nondiabetic bacteremic mice were rescued; however, at the same time, phage GNCP completely failed to protect diabetic bacteremic mice from severe morbidity.

### 3.8. Chronic Studies on STZ Induced Diabetic Mice

Chronic studies of phage for a period of 21 days were carried out on STZ induced mice; results are shown in Table 1.

Phage dose of 3 × 10^9^ PFU/mL significantly reduced to 95.19 ± 4.15 mg/dL from 121.97 ± 4.15 mg/dL. However, at day 7 and day 15 it reduced moderately to 161.21 ± 4.25 mg/dL and 121.97 ± 4.15 mg/dL, respectively, as compared with the diabetic group.

The group treated with standard drug, glipizide, showed a maximum reduction in diabetic blood glucose levels. Significant reduction to 189.080 ± 8.610 mg/dL and 128.87 ± 9.83 mg/dL was noted on the 7th and 15th days of treatment, respectively, while further reduction to 105.79 ± 5.9 blood glucose level was noted on the 21st day of treatment when compared to the diabetic control group.

### 3.9. The Immune Response to Phage in Diabetic and Nondiabetic Mice

After 28 days of single dose injection of phage GNCP in diabetic and nondiabetic mice, titers of IgG and IgM against the phage increased above the background by 500-fold and 100-fold, respectively, in both groups. No substantial deviation was found between diabetic and nondiabetic IgG and IgM titers against phage GNCP. No anaphylactic reactions, changes in core body temperature, or other adverse events were observed in the two groups.

### 3.10. Histopathological Studies

The results of the bacteriophage therapy were also confirmed on the basis of histopathological examination of the vital organs: liver, kidney, lung, and spleen.

**Figure 5:**
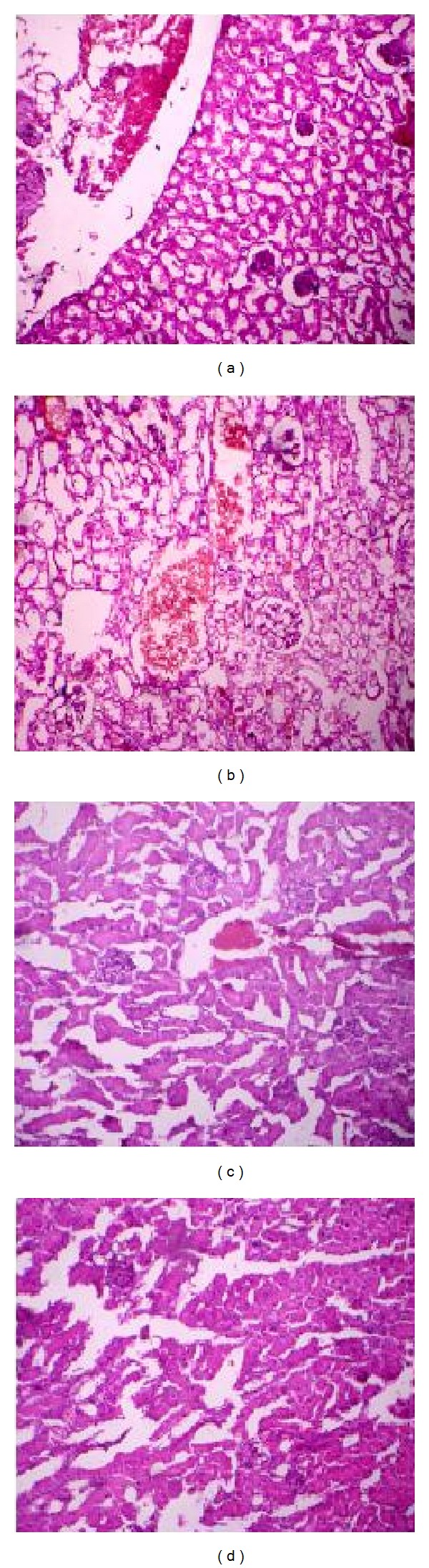
Histopathology of the kidney. Sections of hematoxylin and eosin-stained kidney (×500 magnification) are shown. (a) Diseased mice treated with phage GNCP. (b) Diseased mice treated with imipenem. (c) Diseased mice infected with lethal dose. (d) Control group of mice received PBS.

Histological examination of the normal structure of the kidney of control is illustrated in Figure 5(d). Microscopic examination of the kidney in diseased mice infected with* P. aeruginosa* (Figure 5(c)) revealed some degenerative changes such as prominent internal hemorrhage and atrophied and vacuolated convoluted tubules. Intertubular alterations included the occurrence of collapsed glomeruli and congested glomerular capillaries. Imipenem antibiotic treated mice group (Figure 5(b)) showed focal areas of necrosis, hypertrophied glomeruli, and nuclear degeneration. In the group treated with phage GNCP showed the normal architecture with glomeruli and tubules lined by epithelium with eosinophilic cytoplasm; this showed to be a sign of recovery (Figure 5(a)).

**Figure 6:**
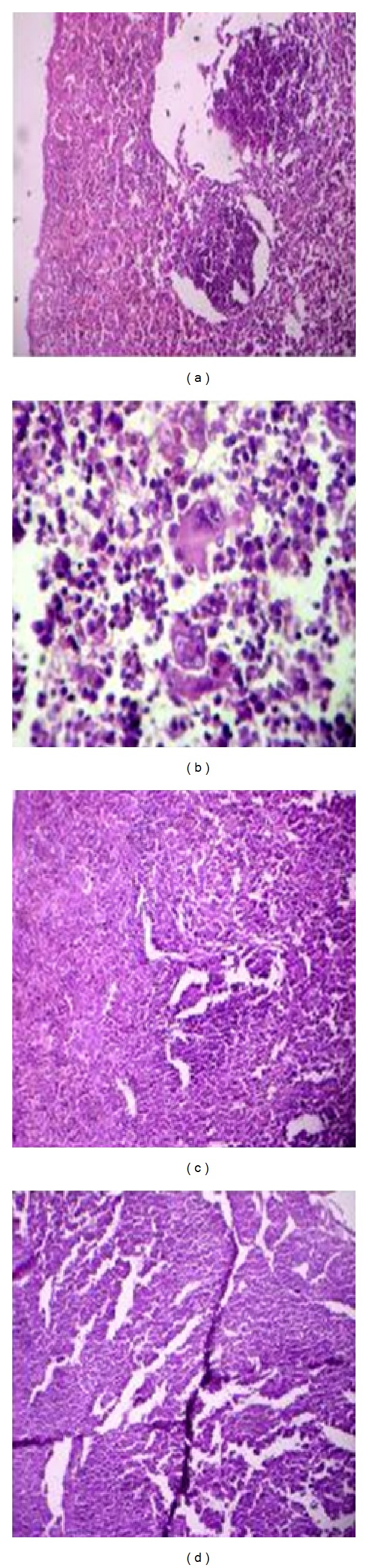
Histopathology of the spleen. Sections of hematoxylin and eosin-stained spleen (×500 magnification) are shown. (a) Diseased mice treated with phage GNCP. (b) Diseased mice treated with imipenem. (c) Diseased mice infected with lethal dose. (d) Control group of mice received PBS.

Histological examination of the normal structure of the spleen of mice in Control group receiving PBS was surround by thick fibrous connective tissue capsule with some myofibroblasts and a covering mesothelium (Figure 6(d)). Internally, thick connective tissue trabeculae bear branches of the splenic artery and veins, with normal white and red pulps. The group of mice that received a lethal dose of* P. aeruginosa *(Figure 6(c)) was represented by internal haemorrhage, complete loss of lymphoid follicular structure, and increased number of megakaryocytes. The imipenem treated group of infected mice revealed thickened splenic capsule with subcapsular dark pigments possibly hemosiderin (Figure 6(b)). The group of infected mice treated with phage showed little expansion of red pulp, and restricted white pulps were detected. Vacuolation was also observed. The appearance of degeneration was common all over the spleen tissue with some of the improvement in phage treated mice (Figure 6(a)).

**Figure 7:**
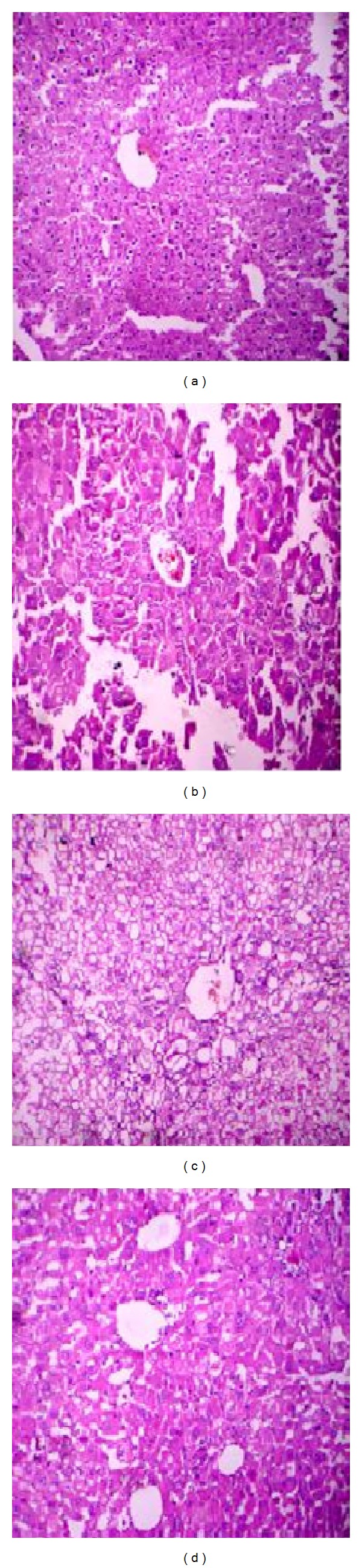
Histopathology of the liver. Sections of hematoxylin and eosin-stained liver (×500 magnification) are shown. (a) Diseased mice treated with phage GNCP. (b) Diseased mice treated with imipenem. (c) Diseased mice infected with lethal dose. (d) Control group of mice received PBS.

Histological examination of the group showed normal architecture with occasional dilated central vein with feathery degeneration of hepatocytes (Figure 7(d)). The section of diseased mice showed degeneration of hepatocytes with focal areas of hemorrhages (Figure 7(c)). The segment of the liver from the antibiotic treated mice showed eccentrically placed nuclei with vacuolated cytoplasm and few of hepatocytes showing feathery degeneration of hepatocytes with focal areas of hemorrhage (Figure 7(b)). The phage treated mice group showed the normal architecture with little quantity of regeneration of focal areas of hemorrhages (Figure 7(a)).

**Figure 8:**
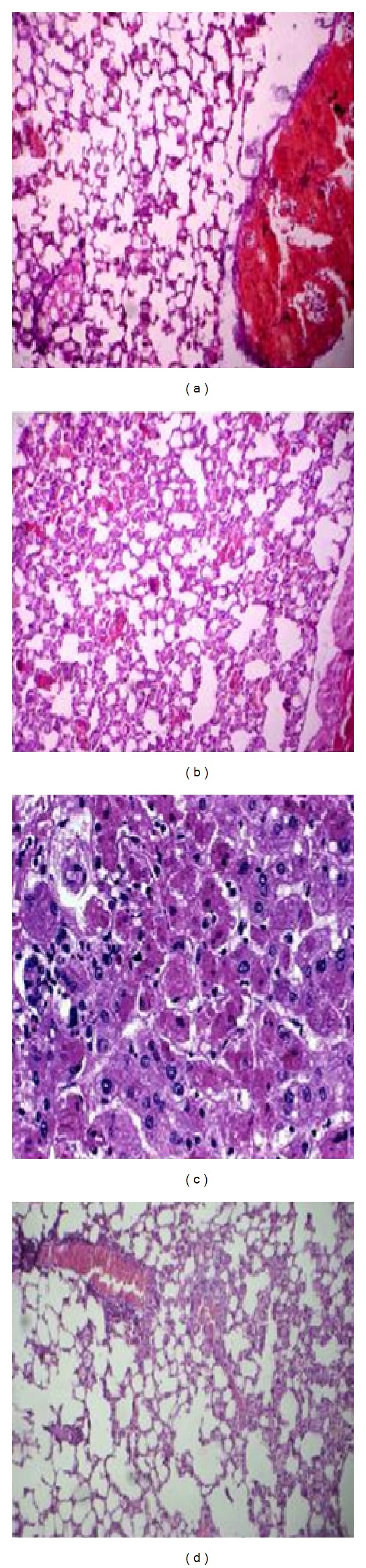
Histopathology of the lung. Sections of hematoxylin and eosin-stained lung (×500 magnification) are shown. (a) Diseased mice treated with phage GNCP. (b) Diseased mice treated with imipenem. (c) Diseased mice infected with lethal dose. (d) Control group of mice received PBS.

Histological examination of the normal structure of the lung from control group section studied shows aerated alveolar spaces and few dilated and congested blood vessels (Figure 8(d)). Microscopy examination of the lung in diseased mice infected with* P. aeruginosa *(Figure 8(c)) revealed significant inflammatory lung disease; many medium and small airways obstructed with beads and localized cell inflammation were observed. The beads were still visible in some sections, suggesting that the infection was sustained antibiotic treated group of mice Figure 8(b). In the group treated with phage GNCP (Figure 8(a)) showed the normal architecture with aerated alveolar spaces and few dilated and congested blood vessels with no signs of damaged.

## 4. Discussion

*P. aeruginosa *is an important opportunistic pathogen that causes chronic infections in the lungs of patients with the genetic disease cystic fibrosis and acute infections such as severe skin infections in cases of burns and urinary tract infections in immunocompromised individuals [11].* P. aeruginosa* is the one among the dominant pathogen, particularly in diabetic patients, causing a range of diseases like foot infections, pneumonia, postoperative infections, UTI, and invasive external otitis [12].

To deduce the severity of infection due to* P. aeruginosa *in the immunocompromised patients, an experimental model of STZ induced diabetic mice with bacteremia was developed. Experimental diabetes was induced by multiple doses of STZ [150 mg (kg^−1^ body weight)] in a mouse model. Animals with blood glucose levels >250 mg/dL were considered a diabetic model. The STZ diabetic model can serve as a better model since diabetes may be initiated at a younger age when all animals have reached maturity and with negligible weight loss [13, 14].

In this study, 12* P. aeruginosa* isolates were isolated from 120 diabetic clinical samples, and most of them were multidrug resistant including imipenem. DPA-12 was selected for further studies based on its susceptibility to phage and drug resistance.

Experimental induction of bacteremia with* P. aeruginosa *DPA-12 was exacerbated in diabetic mice compared with nondiabetic mice. 100% mortality was observed in both groups. However, 100% mortality occurred in diabetic mice within 36 h, in contrast to 100% in nondiabetic mice at 48 h. The severe morbidity and higher mortality rate in diabetic bacteremic mice than in nondiabetic bacteremic mice suggested that diabetic animals are prone to bacterial infections, including* P. aeruginosa *[15, 16].

The effect of imipenem on* P. aeruginosa *bacteremic diabetic and nondiabetic mice revealed more greatly enhanced clearance of* P. aeruginosa *from the 20% of nondiabetic mice than from diabetic bacteremic mice. However, mice treated with multiple imipenem injections showed a survival rate, suggesting that the failure or poor efficacy of imipenem treatment in diabetic mice correlates with the fact that diabetic patients with bacterial infections need to undergo longer antibiotic treatment [17]. The present study provides the first experimental evidence that administration of imipenem is poorly protective of diabetic bacteremic mice compared to nondiabetic bacteremic mice under conditions of* P. aeruginosa *lethal bacteremia used in our work.

Although antibiotics have historically been successful for the treatment of wound infections, the emergence of MDR bacteria and the failure of drug discovery programs over the last 10 years to provide new broad spectrum antibiotics with truly novel modes of action pose a major threat to public health worldwide. In this light, the western critical skepticism towards phage therapy was once more accompanied by a renewed interest and reappraisal of the beginning of the 21st century [18]. The innocuous nature of phage was demonstrated by adding high- titer T4 phage stock to the drinking water of human volunteers [19]. Various research groups performed controlled animal experiments and reported the effectiveness of phage therapy for treatment of vancomycin-resistant* Enterococcus faecium* [6] and methicillin-resistant* Staphylococcus aureus* infections [20].

Many research groups turned their attention to the phage treatment of* P. aeruginosa *infections in mice, guinea pigs, and pet dogs [21]. A report on the treatment of single cases of human burns, wounds indicates that bacteriophage multiplication is associated with clinical improvement.

A first randomized, double-blind, and placebo-controlled phase I/II clinical trial was executed on 24 patients suffering from chronic otitis caused by MDR* P. aeruginosa*, showing efficacy and safety in the treatment of this infection [22]. An important step was recently taken by the detailed description of a quality-controlled small-scale production of a bacteriophage preparation, leading to a safety trial in burn wound patients at the Burn Centre of the Queen Astrid Military Hospital in Brussels, Belgium [23]. Nevertheless, a crucial condition towards practical application of phage therapy will be the availability of a large library of well-characterized phage, subjected to in-depth genomic analysis to confirm the absence of undesired genes [24].

Hence, in the present work, an observational evaluation of the healing potential of newly isolated bacteriophage in diabetic and nondiabetic mice was performed. The phage isolated was found to form plaques on three MDR* P. aeruginosa *clinical isolates.* In vitro *characterization of phage showed it was able to multiply very rapidly on a* P. aeruginosa *culture DPA-12, reaching counts of 10^8^ PFU/mL within 2 h at 37°C. Electron microscopy revealed that phage GNCP has an icosahedral head, approximately 50 nm, and a nonrigid tail. Based on the morphology the phage is tentatively placed in the Siphoviridae family [25]. The phage DNA was 21kb length and was found resistant with several restriction endonucleases.

The i.p. administration of purified phage GNCP rescued both diabetic and nondiabetic mice from* P. aeruginosa *lethal bacteremia. A single i.p. injection of 3 × 10^9^ PFU phage GNCP showed efficient protection in both diabetic (90%) and nondiabetic (100%) bacteremic mice (Figures 3(a) and 3(b)). The combination of diabetic complications and the heavy bacterial load might have restricted phage GNCP activity to 90% protection in diabetic bacteremic mice. The same effectiveness of phage was attained even when treatment was delayed up to 4 h in both diabetic and nondiabetic bacteremic mice, whereas the protection rate was reduced in diabetic mice when phage GNCP was administered after 4 and 6 h of lethal bacterial challenge. In contrast, nondiabetic bacteremic mice were rescued even when treatment was delayed up to 20 h after lethal bacterial challenge.

These experiments demonstrate a powerful curative effect of phage on IMP R-Pa bacteremia in our mouse model. The survival rate between phage treated and the control groups is statistically significantly different. The survival rate of bacteremic mice by delaying treatment was reduced, suggesting that stressed animals are more sensitive to various factors; in this case either the phage itself or trace amounts of endotoxins and exotoxins are present in the phage preparations. Healthy animals did not display apparent reactions to these factors, as evidenced by the lack of any adverse effects in the control groups inoculated with a high dose of the phage preparation.

From the results of chronic study on STZ induced diabetes in mice on 21st, it was found that the phage dose (3 × 10^9^ PFU/mL) has reduced the blood glucose levels from 263.81 ± 6.679 mg/dL to almost normal levels of 95.19 ± 4.15 mg/dL which was more significant compared to standard glipizide (105.79 ± 5.9 blood glucose level) than compared to the diabetic control group.

The most significant finding of this work lay in the comparison of the outcome of treatment of diabetic and nondiabetic bacteremic mice with imipenem and phage GNCP. Evaluation of results confirmed that a single intraperitoneal injection of the phage dose was more efficacious than the multiple doses of imipenem. The reduction in bacterial load was reflected in the lower morbidity and mortality observed in the phage-GNCP-treated group. These results also agree with earlier studies which showed a marked difference in the effect of phage therapy was observed in groups treated with bacteriophage compared to antibiotics [26–28]. It is well known that drugs are catabolized and removed from the body (half life span), whereas phage keeps on multiplying until all host bacteria are followed in the circulation and killed like guided targets. It has been reported that a small population of mutant I phage survived in the circulation, with a concomitant alteration to major head protein E [8]. Therefore, the authors postulated that such “serially passed” immune-escape mutants may facilitate improvements in the therapeutic efficacy of phage.

The present study showed that phage GNCP induces an immune response in both diabetic and nondiabetic mice; the levels of IgG (500-fold) and IgM (100-fold) in both diabetic and nondiabetic mice revealed that the immune response to phage GNCP was not associated with anaphylaxis or other adverse immunological reactions. Similar findings were reported in the treatment of* P. aeruginosa *cystic fibrosis strains [29]. Histological analysis confirmed that the organ damage in the treated group was less severe than in the antibiotic and untreated animals. Bacteria were detected causing severe impairment in most critical organs, especially the liver and spleen in the mice treated with antibiotic and untreated animals. In contrast, the bacteriophage treated mice showed moderate or mild impairment of vital organs observed. No alterations were observed in uninfected animals. These observations were consistent with the fact that bacteriophage targets extracellular bacteria and also the role of phagocytosis in bacterial removal [30].

Based on our observations of this study, phage therapy is used as an alternative therapy for the patients not responding to multidrug resistance regime.

## Figures and Tables

**Table 1:** Chronic study of phage (3 × 10^9^) on blood glucose level in STZ induced diabetic mice.

Groups	Blood glucose level mg/dL			
	0	7	15	21
Control +ve (diabetic normal)	240.20 ± 5.167	273.018 ± 6.197	286.9 ± 619	262.29 ± 6.46
Standard glipizide 10 mg/kg	258.00 ± 4.496	189.080 ± 8.610**	128.87 ± 9.83**	105.79 ± 5.9**
Phage (3 × 10^9^)	263.081 ± 6.679	161.21 ± 4.25**	121.97 ± 4.15**	95.19 ± 4.15**

Values are mean ± SEM; *n* = 6  *P < 0.05, **P < 0.01.

*Indicates level of significance.
